# Cumulative Lactation and Clinical Metabolic Outcomes at Mid-Life among Women with a History of Gestational Diabetes

**DOI:** 10.3390/nu14030650

**Published:** 2022-02-03

**Authors:** Pandora L. Wander, Stefanie N. Hinkle, Daniel A. Enquobahrie, Jing Wu, Sylvia H. Ley, Louise G. Grunnet, Jorge E. Chavarro, Mengying Li, Anne A. Bjerregaard, Aiyi Liu, Peter Damm, Seth Sherman, Shristi Rawal, Yeyi Zhu, Liwei Chen, James L. Mills, Frank B. Hu, Allan Vaag, Sjurdur F. Olsen, Cuilin Zhang

**Affiliations:** 1 Veterans Affairs Puget Sound Health Care System, Seattle, WA 98108, USA; 2 Department of Medicine, University of Washington, Seattle, WA 98195, USA; 3 Department of Biostatistics, Epidemiology, and Informatics, Perelman School of Medicine, University of Pennsylvania, Philadelphia, PA 19104, USA; Stefanie.Hinkle@Pennmedicine.upenn.edu; 4 Department of Epidemiology, University of Washington, Seattle, WA 98195, USA; danenq@u.washington.edu; 5 Glotech, Rockville, MD 20850, USA; jing.wu2@nih.gov; 6 Department of Epidemiology, School of Public Health and Tropical Medicine, Tulane University, New Orleans, LA 70118, USA; sley@tulane.edu; 7 Department of Nutrition, Harvard T.H. Chan School of Public Health, Boston, MA 02115, USA; jchavarr@hsph.harvard.edu (J.E.C.); nhbfh@channing.harvard.edu (F.B.H.); 8 Steno Diabetes Center, 2730 Copenhagen, Denmark; louise.groth.grunnet.02@regionh.dk (L.G.G.); allan.arthur.vaag@regionh.dk (A.V.); 9 Department of Epidemiology, Harvard T.H. Chan School of Public Health, Boston, MA 02115, USA; 10 Division of Intramural Population Health Research, Eunice Kennedy Shriver National Institute of Child Health and Human Development, National Institutes of Health, Bethesda, MD 20892, USA; mengying.li@nih.gov (M.L.); liua@mail.nih.gov (A.L.); millsj@exchange.nih.gov (J.L.M.); 11 Centre for Fetal Programming, Department of Epidemiology Research, Statens Serum Institute, 2300 Copenhagen, Denmark; anne@ssi.dk (A.A.B.); sfo@ssi.dk (S.F.O.); 12 Center for Clinical Research and Prevention, Bispebjerg and Frederiksberg Hospital, 2400 Frederiksberg, Denmark; 13 Center for Pregnant Women with Diabetes, Department of Obstetrics, 2100 Copenhagen, Denmark; pdamm@dadlnet.dk; 14 Department of Clinical Medicine, Faculty of Health and Medical Sciences, University of Copenhagen, 1165 Copenhagen, Denmark; 15 The EMMES Corporation, Rockville, MD 20850, USA; ssherman@emmes.com; 16 Department of Clinical and Preventive Nutritional Sciences, School of Health Professions, Rutgers University, Newark, NJ 08854, USA; shristi.rawal@rutgers.edu; 17 Division of Research, Kaiser Permanente Northern California, Oakland, CA 94611, USA; Yeyi.Zhu@kp.org; 18 Department of Epidemiology and Biostatistics, University of California, San Francisco, CA 94143, USA; 19 Department of Epidemiology, Fielding School of Public Health, University of California, Los Angeles, CA 90095, USA; cliwei86@g.ucla.edu; 20 Channing Division of Network Medicine, Department of Medicine, Brigham and Women’s Hospital and Harvard Medical School, Boston, MA 02115, USA; 21 Department of Obstetrics and Gynaecology, Yong Loo Lin School of Medicine, National University of Singapore, Singapore 119077, Singapore; 22 NUS Bia–Echo Asia Centre for Reproductive Longevity and Equality (ACRLE), Yong Loo Lin School of Medicine, National University of Singapore, Singapore 119077, Singapore

**Keywords:** lactation, breastfeeding, pregnancy, women, diabetes, obesity, biomarkers

## Abstract

Lactation is associated with a lower risk of subsequent cardiometabolic disease among parous women; however, the underlying mechanisms are unknown. Further, the potential protective effects of lactation on cardiometabolic risk markers at mid-life among high-risk women with past gestational diabetes (GDM) are not established. Using data from the Diabetes & Women’s Health Study (2012–2014; *n* = 577), a longitudinal cohort of women with past GDM from the Danish National Birth Cohort (1996–2002), we assessed associations of cumulative lactation duration (none, <6 months, 6–12 months, ≥12–24 months, and ≥24 months) with clinical metabolic outcomes (including type 2 diabetes [T2D], prediabetes, and obesity) and cardiometabolic biomarkers (including biomarkers of glucose/insulin metabolism, fasting lipids, inflammation, and anthropometrics) 9–16 years after enrollment when women were at mid-life. At follow-up, women were 43.9 years old (SD 4.6) with a BMI of 28.7 kg/m^2^ (IQR 24.6, 33.0); 28.6% of participants had T2D, 39.7% had prediabetes, and 41.2% had obesity. Relative risks (95% CI) of T2D for 0–6, 6–12, 12–24, and ≥24 months of cumulative lactation duration compared to none were 0.94 (0.62,1.44), 0.88 (0.59,1.32), 0.73 (0.46,1.17), and 0.71 (0.40,1.27), respectively. Cumulative lactation duration was not significantly associated with any other clinical outcome or continuous biomarker. In this high-risk cohort of middle-aged women with past GDM, T2D, prediabetes, and obesity were common at follow-up, but not associated with history of cumulative lactation duration 9–16 years after the index pregnancy. Further studies in diverse populations among women at mid-age are needed to understand associations of breastfeeding with T2D.

## 1. Introduction

Gestational diabetes (GDM) is prevalent around the world, with the highest burden seen in the Middle East and North Africa, where prevalence estimates, on average, range from 8.4% to 24.5% depending on characteristics of underlying population and diagnostic criteria [1]. Women with past GDM may be unaware of their diagnosis [2]. Women with past GDM are at higher risk of cardiometabolic diseases such as type 2 diabetes (T2D) [3] and cardiovascular disease [4], which they develop at a younger age than parous women without GDM. Among the general population of parous women, longer cumulative duration of lactation is associated with lower risk of T2D [5,6], metabolic syndrome [7], hypertension [6,8], and myocardial infarction [9]. Among women with past GDM, longer duration of lactation has been associated with lower levels of glucose, insulin, and triglycerides and with higher high-density lipoprotein-cholesterol (HDL-c) in the immediate post-partum period [10,11], as well as with lower long-term risk of T2D in large longitudinal cohorts [5,12]. Associations of cumulative lactation duration with cardiometabolic biomarkers at mid-life in women with past GDM, however, are not well characterized. In addition, mechanisms underlying observed associations of lactation duration with cardiometabolic disease are unknown.

Several mechanisms are hypothesized to contribute to a potential protective effect of lactation on cardiometabolic risk including decreased metabolic demand on pancreatic islet cells [13] and more rapid utilization of visceral fat depots and ectopic fat in liver and skeletal muscle [14] during lactation. In addition, higher prolactin levels may directly support islet health and function [13]. Beyond direct effects on glucose and insulin metabolism, differences in lipolysis [15] and favorable changes in lipid metabolism are also present among breastfeeding mothers during the puerperium [10] and persist after weaning [16]. In addition, suckling may lead to higher oxytocin levels [17], with favorable effects on blood pressure regulation [18]. We therefore hypothesized that longer cumulative duration of lactation among women with past GDM is associated with healthier levels of cardiometabolic biomarkers (including measures of glucose/insulin metabolism, fasting lipids, inflammation, and anthropometrics), as well as with lower risk of clinical metabolic outcomes (including hypertriglyceridemia, prediabetes, T2D, and obesity).

## 2. Research Design and Methods

### 2.1. Study Setting and Study Population

The Diabetes & Women’s Health (DWH) Study is a longitudinal cohort study of women with past GDM, identified from the Danish National Birth Cohort (DNBC; 1996–2002) [19], and has been previously described in detail [20]. In Denmark, at the time of the index pregnancy, selective GDM screening was performed among women with risk factors including family history of diabetes, pre-pregnancy overweight or obesity, and age ≥ 35 years, and among parous women with unexplained stillbirth, delivery of infant ≥ 4500 g, or GDM in a past pregnancy [21]. GDM status in DNBC was obtained by review of medical records, International Classification of Diseases codes from the Danish National Patient Register, and/or retrospective interview. In medical records, GDM diagnosis was based on available data such as blood glucose measurements, if available, or on other notes from the doctor indicating GDM. When blood glucose measurements were available, two or more venous glucose values ≥ 6.2 mmol/L (fasting), 10.9 mmol/L (30 min), 11.1 mmol/L (60 min), 9.2 mmol/L (90 min), 8.9 mmol/L (120 min), 8.2 mmol/L (150 min), or 7.3 mmol/L (180 min) were diagnostic of GDM [21,22]. All women diagnosed with GDM in the DNBC (*n* = 1274 out of 91,827 women) were invited to participate in the DWH Study (2012–2014), of whom a total of 607 completed questionnaires and a clinical exam 9–16 years after the index pregnancy complicated by GDM. DWH participants who completed the follow-up clinical exam were similar to the eligible source population in age, pre-pregnancy body mass index (BMI), parity, and smoking status [20]. For this analysis, we excluded women who were diagnosed with type 1 diabetes before the DWH follow-up visit (*n* = 17), were <6 months postpartum (*n* = 7), or were currently lactating (*n* = 12), leaving a final analytic sample of *n* = 577. The study was approved by the Regional Scientific Ethical Committee of the Capital Region of Denmark (record no. H-4-2013-129), and all participants provided written informed consent.

### 2.2. Data Collection

At the DWH Study follow-up visit, women retrospectively reported the duration of lactation for each of their pregnancies. 

Duration of lactation was collected by two methods. First, duration of lactation subsequent to the index pregnancy was collected shortly after the index pregnancy in the DNBC. Then, during the DWH Study follow-up visit, participants completed a questionnaire reporting duration of lactation retrospectively for all pregnancies. The correlation between lactation duration collected after the index pregnancy and lactation duration subsequently recalled for that pregnancy on the DWH Study follow-up questionnaire 9–16 years later was high (Spearman rho = 0.81), with 69.9% of women reporting the duration accurately within a one-month difference [23]. For the current analysis, we calculated cumulative duration of lactation following the index pregnancy by summing the number of lactating months (any lactation) following each birth after the index birth as described in detail previously [23]. Lactation was categorized as: none, <6, 6–12, ≥12–24 months, and ≥24 months.

Outcome measures were collected at the follow-up clinical exam by trained examiners according to a standardized protocol, and consisted of anthropometric measurements, blood pressure measurements, fasting blood samples, and a 2 h, 75 g oral glucose tolerance test (in those without known diabetes). Laboratory methods have been described in detail previously [24]. In brief, blood was collected after an 8–10 h overnight fast and was assayed for glucose (mmol/L), insulin (pmol/L), c-peptide (pmol/L), triglycerides (mg/dL), HDL-c (mg/dL), high-sensitivity C-reactive protein (mg/L), and interleukin-6 (IL-6) level (pg/mL) in plasma using Roche Diagnostics reagents (Indianapolis, IN, USA). Plasma glucose (mmol/L) and insulin (pmol/L) were measured in clinic, while other sample aliquots were processed within 30 min of collection, frozen at −80 °C, and measured at the University of Minnesota. Low-density lipoprotein-cholesterol (LDL-c, mg/dL) was calculated as total cholesterol—HDL-c—(triglycerides/5). C-peptide concentrations were measured using c-peptide microELISA (Quansys Biosciences, Logan, UT, USA; inter-assay coefficient of variation [CV] 10.0% at 3.6 ng/mL and 7.3% at 1.9 ng/mL). Insulin concentrations were measured using a Roche COBAS 6000 chemistry analyzer (Roche Diagnostics, Indianapolis, IN, USA; inter-assay CV 3.1% at 121.2 pmol/L and 3.1% at 377.9 pmol/L). Hemoglobin A1c was measured using a non-porous ion exchange high performance liquid chromatograph assay (Tosoh Automated Analyzer HLC-723G8, Tosoh Bioscience, Inc., San Francisco, CA, USA; inter-assay CV < 1.2%). Height (cm), weight (kg), and waist circumference (WC, cm) midway between the lowest rib and the iliac crest were measured at least twice. Weight change was calculated as the difference between measured weight at the DWH cycle 1 follow up and self-reported pre-pregnancy weight recalled at the first baseline DNBC pregnancy visit. BMI was calculated as kg/m^2^. Mean resting arterial blood pressure was calculated as [(systolic blood pressure) + 2 × (diastolic blood pressure)]/3. The homeostatic model assessment for insulin resistance (HOMA-IR) was calculated as (Fasting Plasma Insulin [mU/L] * Fasting Plasma Glucose [mmol/L])/22.5 and β-cell function (HOMA-B%) as (20 × Fasting Plasma Insulin [mU/L])/(Fasting Plasma Glucose [mmol/L] × 3.5) [25]. For the women who had their follow-up visit at the tertiary care Righospitalet in Copenhagen (*n* = 192), a whole-body dual-energy X-ray absorptiometry (GE Lunar Prodigy, GE Healthcare, Madison, WI, USA) scan was performed. In this subset, abdominal visceral adipose tissue mass was estimated in cm^3^ using enCORE software (version 15, GE Healthcare) v15 [26]. Binary clinical outcomes were defined as obesity (BMI ≥ 30.0 kg/m^2^), hypertriglyceridemia (TG ≥ 200 mg/dL), T2D, a composite endpoint of T2D or prediabetes (impaired fasting glucose or impaired glucose tolerance), and hs-CRP ≥ 3 mg/L [27]. Diagnosis of T2D was based on clinical exam results (A1c ≥ 6.5%, fasting glucose ≥ 7.0 mmol/L, and/or 2 h glucose ≥ 11.1 mmol/L) or self-report of physician diagnosis [28]. Individuals with fasting glucose of 5.6–6.9 mmol/L, 2 h glucose of 7.8–11.1 mmol/L, or A1c 5.7–6.4% were classified as having prediabetes [28]. 

In the DNBC at the index pregnancy, data were collected on age, pre-pregnancy BMI (kg/m^2^) calculated from self-reported pre-pregnancy height and weight, high school education attainment (yes/no), living with partner (yes/no), offspring birthweight (kg), parity prior to the index pregnancy (0, 1, 2, or ≥3), current or former tobacco use (yes/no), alcohol use during pregnancy (yes/no), moderate or vigorous physical activity during pregnancy [metabolic equivalent (MET)-h/week], dietary quality (using a food frequency questionnaire from which the Alternate Healthy Eating Index score [29] was calculated), and pre-pregnancy chronic diseases (yes/no) (including hypertension). At the DWH Study follow-up visit, data were collected on current age, years since most recent pregnancy, total parity (1, 2, or ≥3), menopausal status, and prevalent chronic diseases (yes/no, including self-reported heart disease, T2D, cancer, gout, elevated cholesterol, and elevated blood pressure), recent medication use including the use of antidiabetic medications, and family history of diabetes in a first-degree relative. Data on race/ethnicity are not available, but roughly 90% of the Danish female population during the DWH study period reported at least one parent of Danish ancestry [30].

### 2.3. Statistical Analyses

We estimated the mean, median, or frequency, as applicable, of covariates within each category of cumulative lactation duration. Statistical significance was estimated using ANOVA, Kruskal–Wallis tests, or chi-square tests, respectively. Regression models for continuous outcomes used log-transformed outcomes, and the final results were presented as the percent difference in each outcome (weight change, A1c, fasting glucose, fasting insulin, fasting c-peptide, HOMA-IR, HOMA-%β, fasting triglycerides, HDL-c, LDL-c, BMI, waist circumference, visceral adipose tissue volume, mean arterial pressure, hs-CRP level, and IL-6 level) comparing each cumulative lactation group to the reference category (no lactation). Models with binary clinical outcomes (TG ≥ 200 mg/dL, T2D, BMI ≥ 30 kg/m^2^, and hs-CRP ≥ 3 mg/L, and the composite endpoint of T2D or prediabetes) utilized modified Poisson regression with robust variance to estimate relative risks (RRs) and 95% confidence intervals (95% CI) for each category of cumulative lactation duration. Multiple imputation with 20 replicates was used to address missing exposure and covariate data [31]. We assumed data were missing at random. The missing data rate for the most commonly missing imputed variable (high school education attainment) was 26%. Each missing variable was imputed using lactation and covariate data at both time points (i.e., pregnancy and follow-up) and the log-transformed continuous outcomes. The distributions of the imputed variables were checked, and the range of the continuous values was limited to the range in the original data. Models were adjusted for variables based on prospective assessment prior to or during the index pregnancy including pre-pregnancy chronic disease, age at index pregnancy, pre-pregnancy BMI, living with partner, high school education or greater, current or former tobacco use, alcohol use during the index pregnancy, Alternate Healthy Eating Index score, moderate-vigorous physical activity MET-h/wk, family history of T2D, and parity at the time of the index pregnancy, as well as the number of pregnancies for which cumulative lactation was reported. Sensitivity analyses were performed stratifying by pre-pregnancy BMI (*n* = 220 BMI < 25.0 kg/m^2^ vs. *n* = 312 BMI ≥ 25.0 kg/m^2^). We performed analyses stratified by menopausal status at follow-up *(n* = 492 not post-menopausal, n = 85 post-menopausal). We also performed analyses restricted to participants in whom the diagnosis of GDM was confirmed on retrospective records review (*n* = 335), those who reported only one lifetime pregnancy (*n* = 174), those who reported no use of antidiabetic medications in the 48 h prior to the follow-up visit (*n* = 518), and those who did not self-report prevalent chronic disease at follow-up (*n* = 354). All analyses were performed using SAS (version 9.4, SAS Institute) with *p*-values < 0.05 used to determine statistical significance. 

## 3. Results

On average, women were 31.7 (± 4.4) years old (mean (± SD)) at the index pregnancy and had a pre-pregnancy BMI of 27.3 (± 5.7) kg/m^2^ (Table 1). Women in categories of longer cumulative lactation duration had lower pre-pregnancy BMI, greater parity, and a shorter time interval between the last pregnancy and the DWH follow-up visit. At the DWH Study follow-up visit, women were 43.9 (± 4.6) years old with BMI of 28.7 (24.6, 33.0) kg/m^2^; 41.2% had obesity (BMI ≥ 30 kg/m^2^), 14.7% were post-menopausal, 28.6% had T2D, and 39.7% had either prediabetes or T2D (Table 2). Women in categories of longer cumulative lactation duration had lower waist circumference and BMI at follow-up.

After adjustment, cumulative lactation duration was not associated with any clinical outcome or continuous biomarker. For T2D, RR (95% CIs) by increasing categories of cumulative lactation duration compared to none were 0.94 (0.62, 1.44), 0.88 (0.59, 1.32), 0.73 (0.46, 1.17), and 0.71 (0.40, 1.27). RR for other binary outcomes in relation to categories of cumulative lactation duration are shown in Table 3**.** For A1c, % differences (95% CI) by increasing categories of cumulative lactation duration compared to none were −2.7% (−6.2, 1.0), −2.1% (−5.4, 1.4), −2.5% (−6.0, 1.1), and −4.1% (−8.3, 0.2). For fasting glucose, % differences (95% CI) were −1.9% (−8.0, 4.7), 0.2% (−5.7, 6.5), −2.2% (−8.3, 4.3), and −4.3% (−11.5, 3.5) respectively. Percent difference for other continuous outcomes in relation to categories of cumulative lactation duration are shown in Table 4. 

For analyses stratified by pre-pregnancy BMI, results for the continuous outcomes were similar to the overall results and the same was true for the binary clinical outcomes among women with pre-pregnancy BMI ≥ 25 kg/m^2^ (Supplementary Table S1). Very few adverse binary outcomes were reported among women with pre-pregnancy BMI < 25 kg/m^2^; therefore, these models did not converge. There were some statistically significant results that differed from the overall results in the analyses stratified by menopausal status, but the 95% CI were very wide. Specifically, among post-menopausal women, IL-6 and HDL had a significant (inverse) association with cumulative lactation duration (Supplementary Table S2). For analyses restricted to women with a confirmed diagnosis of GDM, women who reported only one lifetime pregnancy, women who reported no use of antidiabetic medications in the 48 h prior to the follow-up visit, and women without self-reported prevalent chronic disease at follow-up, results were similar to the overall results (data not shown).

## 4. Discussion

In this well-characterized cohort of Danish women with past GDM, we found a high prevalence of T2D (28.6%), prediabetes or T2D (39.7%), and obesity (41.2%) at mid-life (9–16 years after the index pregnancy, mean age 44 years). After adjustment for maternal risk factors including pre-pregnancy BMI at the index pregnancy, parity, and lifestyle factors, longer duration of lactation was not associated with any of the cardiometabolic biomarkers or clinical metabolic outcomes that we assessed compared to no lactation. To our knowledge, this study is the first to systematically assess associations of lactation duration with a broad variety of cardiometabolic biomarkers among women at mid-life.

In prior studies of women with past GDM, longer duration of lactation has been associated with lower levels of glucose, insulin, and triglycerides, with higher HDL-c in the immediate post-partum period [10,11], and with lower 2-year incidence of T2D [32]. Three studies have also demonstrated a long-term inverse association of duration of lactation with development of T2D in later life among women with GDM over up to 30 years of follow-up [5,12]. An analysis of Coronary Artery Risk Development in Young Adults (CARDIA) participants demonstrated graded inverse associations of lactation duration with incident T2D in a cohort of black and white U.S. women (including 155 with GDM) over 30 years of follow-up with no evidence of effect modification by GDM status [5]. Second, an analysis of data from a German cohort (the German GDM Study) demonstrated a 10-year longer median diabetes-free duration among women who lactated for any duration compared to women who reported no lactation over 19 years of follow-up [12]. Finally, a recent analysis by our group [33] among Nurses’ Health Study (NHS) II participants—who had a median age of 58 years—demonstrated an inverse association of lactation duration with fasting insulin and C-peptide (*n* = 543), and a lower risk of T2D (*n* = 4372) over 25 years of follow-up. In contrast, similar to the null findings in the present study, in an earlier study in the subgroup of women with past GDM in the NHS II, duration of lactation was not associated with subsequent T2D (HR 0.96) at mid-life (mean age, 12 years after the index pregnancy) [34]. 

The current findings, which are generally null, may at first glance appear to contradict previous reports including our own recent findings in the NHS II cohort, but there are notable differences between the study populations that may contribute to or partially explain the differences seen. These include: (1) differences in duration of follow-up, which ranged from 9–16 years in the current study to 30 years in Gunderson et al. [5]; (2) post-menopausal status at follow-up, which was not reported in all studies, but was only 14.7% in our population, which was comparatively younger than the other groups studied; and (3) the cumulative incidence of diabetes, which ranged from an estimated ~10% over 12 years in Stuebe et al. [34] to >48% over 19 years in Ziegler et al. [12] (with a cumulative incidence of 28.6%, the current study fell in the middle). In light of the substantial differences in cumulative diabetes incidence across these cohorts, the current findings might suggest that the protective association of lactation with cardiometabolic disease may depend on other maternal or pregnancy-related factors. One such factor may be severity of dysglycemia during pregnancy. The studies cited above used a variety of cutpoints for GDM diagnosis, which may have resulted in the inclusion of women with relatively more severe dysglycemia during pregnancy in our cohort compared to others. For the current cohort, cutpoints three standard deviations above mean levels in a cohort of 40 healthy Danish women were used to define diabetes [35]. These values were relatively higher than the other cohorts, which primary used Carpenter–Coustan or National Diabetes Data Group criteria. The lack of a protective effect from lactation in our cohort in contrast to the other populations studied suggests the possibility that, among women who exhibit more severe dysglycemia in pregnancy, the disease may have progressed too far for lactation to exert a protective long-term effect. Consistent with this hypothesis, in the German GDM Study, Ziegler et al. reported that the protective effect of lactation was not present in women who required insulin during pregnancy (HR 0.81, *p* = 0.4) compared to those with diet-controlled GDM (HR 0.46, *p* = 0.014) [12], suggesting that the severity of insulin resistance or β-cell dysfunction during pregnancy might modify the relationship between lactation duration and progression of dysglycemia. Studies assessing whether severity of GDM or the use of different GDM diagnostic criteria modifies the association of lactation duration with subsequent T2D could help improve understanding of the mechanisms by which lactation might protect against the development of T2D and/or inform tailored strategies for peripartum counseling. 

Major strengths of this study include the hybrid longitudinal study design combining DNBC pregnancy data with follow-up data from the DWH Study, which provided an opportunity to follow a large, high-risk cohort of women for more than a decade and characterize modifiable risk factors associated with an extensive list of cardiometabolic outcomes. Limitations include the retrospective ascertainment of cumulative lactation duration; however, correlation between lactation duration for the index pregnancy collected shortly afterward and lactation duration recalled for that pregnancy on the DWH follow-up questionnaire 9–16 years later was high (Spearman rho = 0.81), with 69.9% of women reporting the duration accurately within 1 month [23]. Secondly, deaths were not reported, but the mortality rate among women in this age group in Denmark was 52.58/100,000 in 2014 [36]; therefore, the expected number of deaths in this cohort of 577 women over 16 years would be <1. Lastly, because of factors such as the racial/ethnic homogeneity of the cohort, the stringent criteria used for GDM diagnosis, and the selective approach used for GDM screening, the findings may not be generalizable to other populations of parous women with past GDM; however, the relative homogeneity of the study population reduces the potential for unmeasured confounding by socio-economic status.

In conclusion, in our cohort of women with past GDM, we found no significant evidence of a protective effect of lactation on the development of T2D or associated cardiometabolic risk markers 9–16 years after the index pregnancy when women were at mid-life. The high prevalence of T2D, prediabetes, and obesity in this cohort at mid-life highlights the importance of longitudinal preventive care and screening among women with past GDM, who frequently fail to receive post-partum glucose tolerance testing [37]. Further studies in diverse populations among women at mid-age are needed to understand associations of breastfeeding with T2D.

## Figures and Tables

**Table 1:** Characteristics of women with past gestational diabetes mellitus enrolled in the Diabetes & Women’s Health Study, overall and stratified by cumulative duration of lactation.

		Cumulative Lactation Duration, Months					
	Overall	None	>0 to <6	6 to <12	12 to <24	≥24	*p* for Difference **
	*n* = 577 *	*n* = 57	*n* = 101	*n* = 171	*n* = 143	*n*= 60	
**Characteristics at Index Pregnancy**							
Age, years	31.7 (4.4)	31.1 (4.3)	31.9 (4.0)	32.4 (4.7)	30.9 (4.2)	31.2 (4.5)	0.034
Pre-pregnancy BMI, kg/m^2^	27.3 (5.7)	29.7 (6.4)	28.7 (5.6)	26.9 (5.5)	25.8 (5.0)	26.5 (5.8)	<0.0001
Pre-pregnancy BMI by category							0.0002
<25.0	220 (38.1)	12 (21.1)	28 (27.7)	69 (40.4)	68 (47.6)	28 (46.7)	
25.0–29.9	258 (27.4)	15 (26.3)	24 (23.8)	49 (28.7)	38 (26.6)	17 (28.3)	
≥30.0	154 (26.7)	24 (42.1)	40 (39.6)	41 (24.0)	26 (18.2)	11 (18.3)	
Alternate Healthy Eating Index	48.5 (7.6)	45.5 (6.9)	48.3 (8.5)	49.1 (6.9)	49.1 (6.9)	50.1 (7.5)	0.039
Moderate-vigorous physical activity, MET-h/wk	0 (0, 30)	0 (0, 0)	0 (0, 30)	0 (0, 45)	0 (0, 45)	0 (0, 45)	0.630
Offspring birthweight, kg	3.8 (0.6)	3.7 (0.8)	3.8 (0.7)	3.8 (0.6)	3.8 (0.5)	3.7 (0.5)	0.353
GDM case verified, y	335 (58.1)	35 (61.4)	65 (64.4)	111 (64.9)	71 (49.7)	31 (51.7)	0.122
Living with partner, y	514 (89.1)	43 (75.4)	86 (85.2)	161 (94.2)	132 (92.3)	56 (93.3)	0.848
High school education or greater, y	174 (30.2)	15 (26.3)	26 (25.7)	48 (28.1)	54 (37.8)	23 (38.3)	0.053
Parity at the index pregnancy							0.0010
0	208 (36.1)	29 (50.9)	33 (32.7)	41 (24.0)	68 (47.6)	28 (46.7)	
1	210 (36.4)	16 (28.1)	36 (35.6)	75 (43.9)	45 (31.5)	18 (30.0)	
2	92 (15.9)	4 (7.0)	17 (16.8)	35 (20.5)	20 (14.0)	7 (11.7)	
≥3	35 (6.1)	2 (3.5)	7 (6.9)	14 (8.2)	4 (2.8)	4 (6.7)	
Pre-pregnancy chronic disease, y	48 (8.3)	3 (5.3)	11 (10.9)	12 (7.0)	11 (7.7)	3 (5.0)	0.612
Family history of diabetes in a first-degree relative, y	215 (37.3)	24 (42.1)	44 (43.6)	68 (39.8)	49 (34.3)	16 (26.7)	0.193
Smoking in pregnancy, y	149 (25.8)	15 (26.3)	29 (28.7)	44 (25.7)	33 (23.1)	15 (25.0)	0.780
Alcohol use in pregnancy, y	292 (50.6)	21 (36.8)	55 (54.5)	100 (58.5)	77 (53.9)	23 (38.3)	0.035
**Characteristics at 9–16 Year Follow-Up Visit**							
Age, years	43.9 (4.6)	43.1 (4.5)	44.0 (4.3)	44.5 (4.7)	43.2 (4.4)	43.6 (4.4)	0.073
Time since most recent pregnancy, years	10.6 (3.0)	10.3 (3.0)	11.3 (2.3)	11.4 (2.3)	9.6 (2.8)	8.4 (3.1)	<0.0001
Total parity							<0.0001
1	324 (56.2)	38 (66.7)	83 (82.2)	138 (80.7)	46 (32.2)	13 (21.7)	
2	158 (27.4)	14 (24.6)	16 (15.8)	28 (16.4)	76 (53.2)	23 (38.3)	
≥3	57 (9.9)	5 (8.8)	2 (2.0)	5 (2.9)	21 (14.7)	24 (40.0)	
Prevalent chronic disease ***, y	223 (38.7)	25 (43.9)	47 (46.5)	68 (39.8)	44 (30.8)	16 (26.7)	0.031
Prevalent type 2 diabetes, y	114 (19.8)	13 (22.8)	25 (24.8)	38 (22.2)	20 (14.0)	8 (13.3)	0.126
Taken an anti-diabetic medication in the past 48 h, y	59 (10.2)	8 (14.0)	13 (12.9)	20 (11.7)	11 (7.7)	2 (3.3)	0.184
Post-menopausal, y	85 (14.7)	10 (17.5)	16 (15.8)	33 (19.3)	13 (9.1)	6 (10.0)	0.091

Data are presented as mean (standard deviation, SD) or median (interquartile range) for continuous variables and *n* (%) for categorical variables. * Sample size includes *n* = 45 with data missing on duration of lactation. ** *p*-values for global differences in participant characteristics across categories of lactation duration (ANOVA, Kruskal–Wallis, or X2). *** self-reported type 2 diabetes, heart problems, cancer, elevated cholesterol, elevated blood pressure, or gout.

**Table 2:** Cardiometabolic outcomes 9–16 years after the index pregnancy among women with a history of gestational diabetes, overall and stratified by cumulative lactation duration.

		Cumulative Lactation Duration, Months					
	Overall	None	>0 to <6	6 to <12	12 to <24	≥24	
	*n* = 577 *	*n* = 57	*n* = 101	*n* = 171	*n* = 143	*n* = 60	*p*-Value
**Continuous Outcomes**							
Weight change, kg	4.0 (−1.6, 9.8)	2.5 (−4.0, 6.9)	3.8 (−2.8, 9.1)	3.6 (−1.7, 9.4)	4.4 (0.1, 9.5)	7.9 (1.1, 12.5)	0.054
A1c, %	5.4 (5.2, 5.7)	5.5 (5.3, 6.0)	5.4 (5.2, 5.7)	5.4 (5.2, 5.7)	5.3 (5.1, 5.7)	5.4 (5.1, 5.6)	0.003
Fasting glucose, mmol/L	5.5 (5.0, 6.1)	5.4 (5.0, 6.2)	5.6 (5.1, 6.2)	5.5 (5.1, 6.1)	5.4 (5.0, 5.7)	5.3 (4.9, 5.7)	0.051
Fasting insulin, pmol/L	50 (35, 79)	56 (42, 85)	52 (38, 80)	47 (33, 78)	49 (34, 70)	50 (40, 80)	0.257
Fasting c-peptide, pmol/L	983.4 (759.0, 1247.4)	1049.4 (745.8, 1346.4)	1023,0 (798.6, 1234.2)	970.2 (745.8, 1247.4)	924.0 (759.0, 1194.6)	1006.5 (831.6, 1224.3)	0.570
HOMA-IR	1.8 (1.2, 3.0)	1.9 (1.5, 3.9)	1.9 (1.3, 3.0)	1.7 (1.1, 2.8)	1.7 (1.1, 2.8)	1.7 (1.3, 2.6)	0.264
HOMA-%B	72.1 (50.1, 102.1)	78.7 (56.0, 97.1)	75.5 (49.8, 101.8)	65.0 (45.3, 97.5)	71.1 (47.5, 105.3)	84.1 (64.8, 109.2)	0.056
Triglycerides, mg/dL	104 (80, 149)	111 (84, 158)	108 (84, 157)	100 (76, 152)	103 (79, 141)	113 (80, 138)	0.557
HDL-c, mg/dL	57 (48, 68)	54 (43, 68)	55 (46, 63)	57 (48, 70)	58 (48, 68)	57 (49, 70)	0.310
LDL-c, mg/dL	114 (94, 137)	105 (82, 137)	117 (98, 144)	113 (95, 137)	116 (93, 136)	114 (94, 134)	0.304
BMI, kg/m^2^	28.7 (24.6, 33.0)	31.2 (26.3, 35.2)	30.7 (26.6, 34.4)	28.1 (24.1, 32.3)	26.9 (23.7, 30.9)	28.0 (25.3, 32.1)	0.0002
Waist circumference, cm	99.5 (89.4, 109.7)	104.0 (97.9, 115.2)	102.3 (92.4, 113.4)	99.3 (88.8, 109.5)	96.3 (88.2, 105.1)	99.0 (90.4, 106.5)	0.003
Visceral adipose tissue, cm^3^	709 (360, 1272)	1276 (376, 1512)	824 (434, 1471)	597 (353, 1174)	797 (357, 1016)	470 (207, 1272)	0.252
Mean arterial pressure, mm/Hg	91.3 (84.5, 100.7)	96.2 (84.5, 103.7)	91.8 (85.0, 101.2)	91.5 (84.7, 99.8)	89.5 (83.5, 99.5)	90.3 (84.8, 97.1)	0.368
CRP, mg/L	1.6 (0.6, 3.8)	1.8 (0.7, 6.2)	2.2 (1.0, 4.1)	1.2 (0.5, 3.6)	1.4 (0.6, 3.6)	1.4 (0.6, 3.2)	0.039
IL-6, pg/mL	1.3 (0.7, 2.4)	1.4 (0.8, 3.8)	1.4 (0.9, 2.5)	1.3 (0.6, 2.4)	1.2 (0.7, 2.2)	1.2 (0.6, 1.9)	0.217
**Binary Outcomes**							
TG ≥ 200 mg/dL	56 (9.8)	4 (7.0)	12 (12.0)	21 (12.4)	13 (9.2)	2 (3.3)	0.265
Type 2 diabetes	165 (28.6)	21 (36.8)	34 (33.7)	49 (28.7)	30 (21.0)	13 (21.7)	0.073
Prediabetes or T2D	228 (39.7)	27 (47.4)	39 (38.6)	73 (42.9)	46 (32.4)	20 (33.3)	0.184
BMI ≥ 30 kg/m^2^	237 (41.2)	35 (61.4)	54 (53.5)	65 (38.2)	40 (28.0)	21 (35.0)	<0.0001
hs-CRP ≥ 3 mg/L	185 (32.4)	21 (36.8)	38 (38.0)	49 (29.0)	44 (31.4)	16 (26.7)	0.437

Abbreviations: CI (confidence interval), A1c (hemoglobin A1c), HOMA-IR (homeostatic model assessment of insulin resistance), HOMA-%B (homeostatic model assessment of β-cell function), HDL-c (high-density lipoprotein cholesterol), LDL-c (low-density lipoprotein cholesterol), hs-CRP (c-reactive protein), and BMI (body-mass index). Data are presented as mean (SD) or median (IQR) for continuous variables and *n* (%) for categorical variables. *p*-values for parametric continuous variables calculated using ANOVA tests; *p*-values for non-parametric continuous variables calculated using Kruskal–Wallis tests; *p*-values for categorical variables calculated using Chi-square tests. The following definitions were used for the binary outcomes: hypertriglyceridemia, triglycerides ≥ 200 mg/dL; diabetes, A1C ≥ 6.5%, fasting glucose ≥ 7.0 mmol/L, or 2-h oral glucose tolerance test glucose ≥ 11.1 mmol/L or self-report of physician diagnosis; prediabetes or diabetes, fasting glucose ≥ 5.6 mmol/L, 2-h oral glucose tolerance test glucose ≥ 7.8 mmol/L, A1c ≥ 5.7, or self-report of physician diagnosis of diabetes; obesity, BMI ≥ 30.0 kg/m^2^. * Sample size includes *n* = 45 with data missing on duration of lactation.

**Table 3:** Adjusted associations of cumulative lactation duration with dichotomous cardiometabolic outcomes 9–16 years after the index pregnancy among women with a history of gestational diabetes compared to no lactation, *n* = 577.

	Adjusted RR			
	(95% CI)			
Cumulative Lactation Duration, Months	>0 to <6 *n* = 101	6 to <12 *n* = 171	12 to <24 *n* = 143	≥24 *n* = 60
TG ≥ 200mg/dL	1.64	2.15	2.02	0.81
	(0.60, 4.48)	(0.84, 5.47)	(0.77, 5.30)	(0.15, 4.40)
T2D	0.94	0.88	0.73	0.71
	(0.62, 1.44)	(0.59, 1.32)	(0.46, 1.17)	(0.40, 1.27)
Prediabetes or T2D	0.82	0.93	0.78	0.75
	(0.57, 1.18)	(0.67, 1.30)	(0.54, 1.13)	(0.48, 1.19)
BMI ≥ 30 kg/m^2^	1.08	0.89	0.73	0.79
	(0.82, 1.42)	(0.67, 1.19)	(0.52, 1.03)	(0.52, 1.21)
hs-CRP ≥ 3 mg/L	1.16	1.02	1.12	0.97
	(0.77, 1.76)	(0.68, 1.53)	(0.73, 1.70)	(0.54, 1.76)

Abbreviations: CI (confidence interval), TG (triglycerides) T2D (type 2 diabetes), hs-CRP (high-sensitivity c-reactive protein), RR (relative risk), and BMI (body-mass index). The following definitions were used for the binary outcomes: hypertriglyceridemia, triglycerides ≥ 200 mg/dL; diabetes, A1C ≥ 6.5%, fasting glucose ≥ 7.0 mmol/L, or 2-h oral glucose tolerance test glucose ≥ 11.1 mmol/L or self-report of physician diagnosis; prediabetes or diabetes, fasting glucose ≥ 5.6mmol/L, 2-h oral glucose tolerance test glucose ≥ 7.8 mmol/L, A1c ≥ 5.7, or self-report of physician diagnosis of diabetes. Models were adjusted for pre-pregnancy chronic disease, age at index pregnancy, pre-pregnancy body-mass index, living with partner, less than high school education, current or former tobacco use, alcohol use during pregnancy, Alternate Healthy Eating Index score, moderate-vigorous physical activity MET-h/wk, family history of type 2 diabetes, parity at the time of the index pregnancy, and total parity.

**Table 4:** Adjusted associations of cumulative lactation duration with continuous cardiometabolic outcomes 9–16 years after the index pregnancy among women with a history of gestational diabetes compared to no lactation, *n* = 577.

	Adjusted % Difference (95% CI)			
Cumulative Lactation Duration, Months	>0 to <6 *n* = 101	6 to <12 *n* = 171	12 to <24 *n* = 143	≥24 *n* = 60
Weight change, kg	−2.3	−7.3	−8.3	−6.8
	(−12.2, 8.7)	(−16.1, 2.5)	(−17.5, 1.9)	(−18.0, 6.1)
A1c, %	−2.7	−2.1	−2.5	−4.1
	(−6.2, 1.0)	(−5.4, 1.4)	(−6.0, 1.1)	(−8.3, 0.2)
Fasting glucose, mmol/L	−1.9	0.2	-2.2	-4.3
	(−8.0, 4.7)	(−5.7, 6.5)	(−8.3, 4.3)	(−11.5, 3.5)
Fasting insulin, pmol/L	−4.6	−4.6	6.3	19.1
	(−19.5, 13.0)	(-18.7, 11.9)	(-10.1, 25.7)	(-3.1, 46.4)
Fasting c-peptide, pmol/L	0.1	3.4	6.1	15.2
	(−11.3, 13.0)	(−7.8, 15.9)	(−5.9, 19.6)	(−0.6, 33.6)
HOMA-IR	−12.7	−18.2	−4.9	−0.5
	(−32.2, 12.5)	(−35.9, 4.5)	(−27.0, 23.8)	(−27.7, 37.0)
HOMA-%B	1.7	−4.8	7.3	26.4
	(−17.4, 25.1)	(−21.9,16.0)	(−13.5,33.1)	(−2.6,64.0)
Triglycerides, mg/dL	−2.7	−2.2	2.6	2.2
	(−16.1, 13.0)	(−15.0, 12.5)	(−11.4, 18.9)	(−14.6, 22.4)
HDL-c, mg/dL	−2.5	−1.3	−1.4	0.6
	(−9.8, 5.4)	(−8.3, 6.2)	(−8.7, 6.6)	(−8.5, 10.6)
LDL-c, mg/dL	9.6	3	6.6	5.3
	(−1.0, 21.2)	(−6.3, 13.3)	(−3.4, 17.7)	(−6.4, 18.6)
BMI, kg/m^2^	0.8	0.3	−1.3	1.2
	(−3.9, 5.6)	(−4.1, 4.9)	(−5.8, 3.5)	(−4.6, 7.3)
Waist circumference, cm	1	0	−0.3	−0.9
	(−2.3, 4.3)	(−3.0, 3.1)	(−3.4, 3.0)	(−4.8, 3.2)
Visceral adipose tissue, cm^3^	30.4	18.4	29.9	18.4
	(−25.4, 128.0)	(−29.4, 98.7)	(−24.8, 124.4)	(−38.9, 129.6)
Mean arterial pressure, mm/Hg	−2.5	−1.1	−1.6	−1.2
	(−6.1, 1.4)	(−4.7, 2.6)	(−5.3, 2.3)	(−5.7, 3.6)
hs-CRP, mg/L	18	−13.7	13.9	0.2
	(−19.3, 72.5)	(−39.6, 23.4)	(−21.8, 65.9)	(−36.7, 58.5)
IL-6, pg/mL	48.5	19.6	40.6	58.9
	(−61.6, 474.2)	(−66.7, 330.3)	(−63.4, 439.7)	(−68.8, 708.0)

Abbreviations: CI (confidence interval), A1c (hemoglobin A1c), HOMA-IR (homeostatic model assessment of insulin resistance), HOMA-%B (homeostatic model assessment of β-cell function), HDL-c (high-density lipoprotein cholesterol), LDL-c (low-density lipoprotein cholesterol), hs-CRP (high sensitivity c-reactive protein), and BMI (body-mass index). The following definitions were used for the binary outcomes: hypertriglyceridemia, triglycerides ≥ 200 mg/dL; diabetes, A1C ≥ 6.5%, fasting glucose ≥ 7.0 mmol/L, or 2-h oral glucose tolerance test glucose ≥ 11.1 mmol/L or self-report of physician diagnosis; prediabetes or diabetes, fasting glucose ≥ 5.6 mmol/L, 2-h oral glucose tolerance test glucose ≥ 7.8 mmol/L, A1c ≥ 5.7, or self-report of physician diagnosis of diabetes. Models were adjusted for pre-pregnancy chronic disease, age at index pregnancy, pre-pregnancy body-mass index, living with partner, less than high school education, current or former tobacco use, alcohol use during pregnancy, Alternative Healthy Eating Index score, moderate-vigorous physical activity MET-h/wk, family history of type 2 diabetes, parity at the time of the index pregnancy, and total parity.

## Data Availability

The consent given by the participants does not allow for storage of data on an individual level in repositories or journals. Researchers who want access to data sets for replication should submit an application. Access to data sets requires approvals from the Danish Scientific Ethical Committee System, the Danish Data Protection Agency, and the cohort organization.

## Supplementary Materials

The following supporting information can be downloaded at: https://www.mdpi.com/article/10.3390/nu14030650/s1, Table S1. Cumulative duration of lactation and adjusted associations with cardiometabolic outcomes 9–16 years after the index pregnancy, stratified by pre-pregnancy BMI. Table S2: Cumulative duration of lactation and adjusted associations with cardiometabolic outcomes 9–16 years after the index pregnancy, stratified by menopausal status.

## References

[1] Zhu Y., Zhang C. (2016). Prevalence of Gestational Diabetes And Risk Of Progression To Type 2 Diabetes: A Global Perspective. Curr. Diabetes Rep..

[2] Quaresima P., Visconti F., Interlandi F., Puccio L., Caroleo P., Amendola G., Morelli M., Venturella R., Di Carlo C. (2021). Awareness Of Gestational Diabetes Mellitus Foetal-Maternal Risks: An Italian Cohort Study On Pregnant Women. BMC Pregnancy Childbirth.

[3] Bellamy L., Casas J.P., Hingorani A.D., Williams D. (2009). Type 2 Diabetes Mellitus After Gestational Diabetes: A Systematic Review And Meta-Analysis. Lancet.

[4] Tobias D.K., Stuart J.J., Li S., Chavarro J., Rimm E.B., Rich-Edwards J., Hu F.B., Manson J.E., Zhang C. (2017). Association Of History Of Gestational Diabetes With Long-Term Cardiovascular Disease Risk In A Large Prospective Cohort Of Us Women. Jama Intern. Med..

[5] Gunderson E.P., Lewis C.E., Lin Y., Sorel M., Gross M., Sidney S., Jacobs D.R., Shikany J.M., Quesenberry C.P. (2018). Lactation Duration And Progression To Diabetes In Women Across The Childbearing Years: The 30-Year Cardia Study. Jama Intern. Med..

[6] Rameez R.M., Sadana D., Kaur S., Ahmed T., Patel J., Khan M.S., Misbah S., Simonson M.T., Riaz H., Ahmed H.M. (2019). Association Of Maternal Lactation With Diabetes And Hypertension: A Systematic Review And Meta-Analysis. JAMA Netw. Open.

[7] Gunderson E.P., Jacobs D.R., Chiang V., Lewis C.E., Feng J., Quesenberry C.P., Sidney S. (2010). Duration Of Lactation And Incidence Of The Metabolic Syndrome In Women Of Reproductive Age According To Gestational Diabetes Mellitus Status: A 20-Year Prospective Study In Cardia (Coronary Artery Risk Development In Young Adults). Diabetes.

[8] Qu G., Wang L., Tang X., Wu W., Sun Y. (2018). Association Between Duration Of Breastfeeding And Maternal Hypertension: A Systematic Review And Meta-Analysis. Breastfeed Med..

[9] Stuebe A.M., Michels K.B., Willett W.C., Manson J.E., Rexrode K., Rich-Edwards J.W. (2009). Duration Of Lactation And Incidence Of Myocardial Infarction In Middle To Late Adulthood. Am. J. Obstet. Gynecol..

[10] Gunderson E.P., Kim C., Quesenberry C.P., Marcovina S., Walton D., Azevedo R.A., Fox G., Elmasian C., Young S., Salvador N. (2014). Lactation Intensity And Fasting Plasma Lipids, Lipoproteins, Non-Esterified Free Fatty Acids, Leptin And Adiponectin In Postpartum Women With Recent Gestational Diabetes Mellitus: The Swift Cohort. Metab. Clin. Exp..

[11] Gunderson E.P., Hedderson M.M., Chiang V., Crites Y., Walton D., Azevedo R.A., Fox G., Elmasian C., Young S., Salvador N. (2012). Lactation Intensity And Postpartum Maternal Glucose Tolerance And Insulin Resistance In Women With Recent Gdm: The Swift Cohort. Diabetes Care.

[12] Ziegler A.G., Wallner M., Kaiser I., Rossbauer M., Harsunen M.H., Lachmann L., Maier J., Winkler C., Hummel S. (2012). Long-Term Protective Effect of Lactation on The Development of Type 2 Diabetes in Women with Recent Gestational Diabetes Mellitus. Diabetes.

[13] Much D., Beyerlein A., Rossbauer M., Hummel S., Ziegler A.G. (2014). Beneficial Effects of Breastfeeding in Women with Gestational Diabetes Mellitus. Mol. Metab..

[14] Stuebe A.M., Rich-Edwards J.W. (2009). The Reset Hypothesis: Lactation and Maternal Metabolism. Am. J. Perinatol..

[15] Rebuffe-Scrive M., Enk L., Crona N., Lonnroth P., Abrahamsson L., Smith U., Bjorntorp P. (1985). Fat Cell Metabolism in Different Regions in Women. Effect of Menstrual Cycle, Pregnancy, and Lactation. J. Clin. Investig..

[16] Gunderson E.P., Lewis C.E., Wei G.S., Whitmer R.A., Quesenberry C.P., Sidney S. (2007). Lactation and Changes in Maternal Metabolic Risk Factors. Obstet. Gynecol..

[17] Johnston J.M., Amico J.A. (1986). A Prospective Longitudinal Study of the Release of Oxytocin and Prolactin in Response to Infant Suckling in Long Term Lactation. J. Clin. Endocrinol. Metab..

[18] Petersson M., Lundeberg T., Uvnas-Moberg K. (1999). Short-Term Increase and Long-Term Decrease of Blood Pressure in Response to Oxytocin-Potentiating Effect of Female Steroid Hormones. J. Cardiovasc. Pharmacol..

[19] Olsen J., Melbye M., Olsen S.F., Sorensen T.I., Aaby P., Andersen A.M., Taxbol D., Hansen K.D., Juhl M., Schow T.B. (2001). The Danish National Birth Cohort-Its Background, Structure and Aim. Scand. J. Public Health.

[20] Zhang C., Hu F.B., Olsen S.F., Vaag A., Gore-Langton R., Chavarro J.E., Bao W., Yeung E., Bowers K., Grunnet L.G. (2014). Rationale, Design, And Method of the Diabetes & Women’s Health Study—A Study of Long-Term Health Implications of Glucose Intolerance in Pregnancy and Their Determinants. Acta Obs. Gynecol. Scand..

[21] Olsen S.F., Houshmand-Oeregaard A., Granstrom C., Langhoff-Roos J., Damm P., Bech B.H., Vaag A.A., Zhang C. (2017). Diagnosing Gestational Diabetes Mellitus in The Danish National Birth Cohort. Acta Obs. Gynecol Scand..

[22] Grunnet L.G., Hansen S., Hjort L., Madsen C.M., Kampmann F.B., Thuesen A.C.B., Granstromi C., Strom M., Maslova E., Frikke-Schmidt R. (2017). Adiposity, Dysmetabolic Traits, and Earlier Onset of Female Puberty in Adolescent Offspring of Women With Gestational Diabetes Mellitus: A Clinical Study within the Danish National Birth Cohort. Diabetes Care.

[23] Panuganti P.L., Hinkle S.N., Rawal S., Grunnet L.G., Lin Y., Liu A., Thuesen A.C.B., Ley S.H., Olesen S.F., Zhang C. (2018). Lactation Duration and Long-Term Thyroid Function: A Study Among Women with Gestational Diabetes. Nutrients.

[24] Hinkle S.N., Rawal S., Bjerregaard A.A., Halldorsson T.I., Li M., Ley S.H., Wu J., Zhu Y., Chen L., Liu A. (2019). A Prospective Study of Artificially Sweetened Beverage Intake and Cardiometabolic Health among Women at High Risk. Am. J. Clin. Nutr..

[25] Wallace T.M., Levy J.C., Matthews D.R. (2004). Use and Abuse of Homa Modeling. Diabetes Care.

[26] Kaul S., Rothney M.P., Peters D.M., Wacker W.K., Davis C.E., Shapiro M.D., Ergun D.L. (2012). Dual-Energy X-Ray Absorptiometry for Quantification of Visceral Fat. Obesity.

[27] Myers G.L., Rifai N., Tracy R.P., Roberts W.L., Alexander R.W., Biasucci L.M., Catravas J.D., Cole T.G., Cooper G.R., Khan B.V. (2004). Cdc/Aha Workshop on Markers of Inflammation and Cardiovascular Disease: Application to Clinical And Public Health Practice: Report from The Laboratory Science Discussion Group. Circulation.

[28] American Diabetes Association (2017). 2. Classification and Diagnosis of Diabetes. Diabetes Care.

[29] Mccullough M.L., Feskanich D., Stampfer M.J., Giovannucci E.L., Rimm E.B., Hu F.B., Spiegelman D., Hunter D.J., Colditz G.A., Willett W.C. (2002). Diet Quality and Major Chronic Disease Risk in Men and Women: Moving Toward Improved Dietary Guidance. Am. J. Clin. Nutr..

[30] Statistics Denmark. https://Www.Dst.Dk/En/Statistik/Emner/Befolkning-Og-Valg/Indvandrere-Og-Efterkommere/Indvandrere-Og-Efterkommere#.

[31] Zhou X.H., Eckert G.J., Tierney W.M. (2001). Multiple Imputation in Public Health Research. Stat. Med..

[32] Gunderson E.P., Hurston S.R., Ning X., Lo J.C., Crites Y., Walton D., Dewey K.G., Azevedo R.A., Young S., Fox G. (2015). Lactation and Progression to Type 2 Diabetes Mellitus after Gestational Diabetes Mellitus: A Prospective Cohort Study. Ann. Intern. Med..

[33] Ley S.H., Chavarro J.E., Li M., Bao W., Hinkle S.N., Wander P.L., Rich-Edwards J., Olsen S., Vaag A., Damm P. (2020). Lactation Duration and Long-Term Risk for Incident Type 2 Diabetes in Women with a History Of Gestational Diabetes Mellitus. Diabetes Care.

[34] Stuebe A.M., Rich-Edwards J.W., Willett W.C., Manson J.E., Michels K.B. (2005). Duration of Lactation and Incidence of Type 2 Diabetes. JAMA J. Am. Med. Assoc..

[35] Damm P., Handberg A., Kuhl C., Beck-Nielsen H., Molsted-Pedersen L. (1993). Insulin Receptor Binding and Tyrosine Kinase Activity in Skeletal Muscle from Normal Pregnant Women and Women with Gestational Diabetes. Obstet. Gynecol..

[36] Institute for Health Metrics and Evaluation (IHME) Gbd Compare. http://Vizhub.Healthdata.Org/Gbd-Compare.

[37] Quaresima P., Visconti F., Chiefari E., Puccio L., Foti D.P., Venturella R., Vero R., Brunetti A., Di Carlo C. (2018). Barriers to Postpartum Glucose Intolerance Screening in an Italian Population. Int. J. Env. Res. Public Health.

